# Syndromic Recognition of Influenza A Infection in a Low Prevalence Community Setting

**DOI:** 10.1371/journal.pone.0010542

**Published:** 2010-05-07

**Authors:** Po-Yen Huang, Ching-Tai Huang, Kuo-Chien Tsao, Jung-Jr Ye, Shian-Sen Shie, Ming-Yi Yang, Hsieh-Shong Leu, Ping-Cherng Chiang, Yin-Che Weng

**Affiliations:** 1 Division of Infectious Diseases, Department of Medicine, Chang Gung Memorial Hospital, Chang Gung University, Taoyuan, Taiwan; 2 School of Medicine, Chang Gung University, Taoyuan, Taiwan; 3 Department of Laboratory Medicine, Chang Gung Memorial Hospital, Chang Gung University, Taoyuan, Taiwan; 4 Department of Biotechnology and Laboratory Science, Research Center for Emerging Viral Infections, Chang Gung University, Taoyuan, Taiwan; 5 Min-Shen Weng Community Clinic, Taipei, Taiwan; Universidad Peruana Cayetano Heredia, Peru

## Abstract

**Background:**

With epidemics of influenza A virus infection, people and medical professionals are all concerned about symptoms or syndromes that may indicate the infection with influenza A virus.

**Methodology/Principal Findings:**

A prospective study was performed at a community clinic of a metropolitan area. Throat swab was sampled for 3–6 consecutive adult patients with new episode (<3 days) of respiratory tract infection every weekday from Dec. 8, 2005 to Mar. 31, 2006. Demographic data, relevant history, symptoms and signs were recorded. Samples were processed with multiplex real time PCR for 9 common respiratory tract pathogens and by virus culture. Throat swab samples were positive for Influenza A virus with multiplex real time PCR system in 12 of 240 patients. The 12 influenza A positive cases were with more clusters and chills than the other 228. Certain symptoms and syndromes increased the likelihood of influenza A virus infection. The syndrome of high fever plus chills plus cough, better with clustering of cases in household or workplace, is with the highest likelihood (positive likelihood ratio 95; 95% CI 12–750). Absence of both cluster and chills provides moderate evidence against the infection (negative likelihood ratio 0.51; 95% CI 0.29–0.90).

**Conclusions/Significance:**

Syndromic recognition is not diagnostic but is useful for discriminating between influenza A infection and common cold. In addition to relevant travel history, confirmatory molecular test can be applied to subjects with high likelihood when the disease prevalence is low.

## Introduction

The new H1N1 influenza A virus is causing illness in the United States and countries around the world. The significant number of mortality in Mexico foreshadows the horrifying possibility of another deadly pandemic, similar to the Spanish flu of 1918, with huge death toll in young and previously healthy adults. The symptoms include fever, headache, malaise, cough, sore throat, runny nose, myalgia, diarrhea, and vomiting [1]. Many other illnesses can have similar symptoms. People with respiratory tract symptoms are naturally concerned whether they have contracted the virus. Medical professionals have the same concern whether the patients they are seeing are victims of the infection. Although definitions of influenza-like illness have been proposed, it is still not clear whether recognition of a constellation of symptoms or a syndrome, is reliable for clinical diagnosis of influenza infection [2].

A prospective study was conducted in a clinic, to define the role of syndromic recognition for influenza A infection in a community setting. As the majority of people who get upper respiratory tract symptoms prefer to visit community clinics here in Taiwan, we chose such a community clinic for comprehensive sampling of new cases of upper respiratory tract infection.

## Methods

The study was performed at a community clinic of Metropolitan Taipei Area in Taiwan, targeted at adult (over 18 year old) patients who presented a new episode (less than 3 days) of respiratory tract infection. The first consecutive 3 to 6 patients of every weekday were enrolled during the period from Dec. 8, 2005 to Mar. 31, 2006. Under informed consent, throat swab was sampled by a physician and demographic data, relevant history, symptoms and signs were recorded. Prior vaccination specified flu vaccination during the year of 2005. Travel history meant travel abroad during the past 4 weeks. Clusters are family members or colleagues at workplace with similar symptoms. All symptoms were subjective reporting without objective measurement. Signs were documented by the community physicians.

Throat swab was performed with a sterile cotton swab that was then inserted into a vial containing viral transport medium. Samples were transported at 4°C to our clinical microbiology lab at the Chang Gung Memorial Hospital Linkou Medical Center and processed on the same day.

A multiplex real-time PCR (RT-PCR) was developed to detect simultaneously from the same specimen a batch of nine microbial pathogens that are frequently associated with acute respiratory tract infections, including Influenza A virus, Influenza B virus, Parainfluenza virus types 1, 2 and 3, Rhinovirus, Respiratory Syncytial virus, Adenovirus, and Mycoplasma pneumoniae. The specimens were subjected to laboratory examination by both the multiplex RT-PCR and conventional virus culture.

### Ethics Statements

This study was conducted according to the principles expressed in the Declaration of Helsinki and was approved by the Institutional Review Board of Chang Gung Memorial Hospital. All patients provided written informed consent for the collection of samples and subsequent analysis.

### Statistical Analyses

The statistical analyses were performed using a software package (SPSS, version 12.0; SPSS, Inc., Chicago, IL, USA). Continuous variables were compared using Student's t test. Binomial variables were compared using chi-square or Fisher's exact test as appropriate. Variables with a 2-tailed *P* value <0.25 were included in a one-step binary logistic regression analysis to determine the independently-associated factors with laboratory-confirmed cases. The positive likelihood ratio (PLR) and negative likelihood ratio (NLR) were calculated in selected symptoms or symptom complexes. All tests were 2-tailed and considered significant at *P* value <0.05.

## Results

A total of 240 throat swab specimens were collected from adult patients who presented a new episode of respiratory tract infection. Positive results were obtained in 58 (24%) specimens by the multiplex RT-PCR system, while only 12 (5%) specimens were positive by the virus culture (Table 1). No pathogen was detected by either method in 179 (75%) samples.

**Table 1:** Laboratory results for patients with acute respiratory tract symptoms.

	Case Number (Total = 240)		
	Virus Culture	RT-PCR	Overall
Influenza A	5	12	**12**
Influenza B	0	0	**0**
Parainfluenza 1	3	2	**3**
Parainfluenza 2	2	2	**2**
Parainfluenza 3	0	2	**2**
Rhinovirus	0	32	**32**
Respiratory syncytial virus	0	2	**2**
Adenovirus	0	7	**7**
Mycoplasma	N/A*	4	**4**
Enterovirus	2	N/A*	**2**
**Pathogen identified**	**12(5%)**	**58** † **(24%)**	**61** † **(25%)**
**No pathogen identified**	**228(95%)**	**182(76%)**	**179(75%)**

* N/A: not available.

† Five samples were positive for 2 pathogens.

Twelve patients were positive for influenza A virus by RT-PCR system, including 5 positive for influenza A virus culture as well. Demographic data, relevant history, symptoms and signs of these 12 patients were compared to the other 228 patients who revealed negative results for influenza A virus (Table 2). There was no difference in age, sex, flu vaccination, or traveling abroad, but patients with influenza A reported significantly higher percentage of clustering (92% versus 56%, *P* = 0.014). Symptoms with considerable difference were febrile sensation, high fever, chilly sensation and chills. Surprisingly there was no difference with regard to other systemic symptoms believed to be more prominent with influenza infection, including headache, myalgia, and malaise. Sorethroat was one of the symptoms in some definitions of influenza like illness [3], [4], but this is not characteristic for our influenza cases. There was no patient with either tonsillar exudates or neck lymphadenopathy. Multivariate analysis of this comparison revealed clusters (OR: 9.7; 95% CI: 1.1–85; *P* = 0.039) and chills (OR 12; 95% CI: 1.8–83; P = 0.010) as the 2 independently significant features for influenza A infection.

**Table 2:** Demographic data, history, symptoms and signs of patients with acute respiratory tract symptoms.

	Influenza A (n = 12)	Others (n = 228)	*P* value
**Demography**			
Age (mean±SD)	41±11	39±12	0.568
Sex, male	25% (n = 3)	81% (n = 36)	0.549
**History**			
Prior vaccination	17% (n = 2)	16% (n = 37)	1.000
Travel history	17% (n = 2)	8·3% (n = 19)	0.283
Clusters*, †	92% (n = 11)	56% (n = 127)	0.014
**Symptoms**			
Febrile sensation*	75% (n = 9)	40% (n = 91)	0.031
High fever*	42% (n = 5)	15% (n = 33)	0.026
Chilly sensation*	67% (n = 8)	24% (n = 55)	0.003
Chills*, †	50% (n = 6)	5·3% (n = 12)	<0.001
Headache*	67% (n = 8)	47% (n = 108)	0.192
Myalgia	67% (n = 8)	53% (n = 120)	0.342
Malaise	75% (n = 9)	67% (n = 153)	0.756
Sorethroat	75% (n = 9)	74% (n = 168)	1.000
Cough	83% (n = 10)	65% (n = 149)	0.347
Nasal congestion	50% (n = 6)	66% (n = 151)	0.350
Rhinorrhea	75% (n = 9)	63% (n = 143)	0.543
Sneezing	58% (n = 7)	58% (n = 133)	1.000
**Signs**			
Exudative tonsils	0	0	1.000
Neck lymphadenopathy	0	0	1.000

* Variables were included in a binary logistic regression analysis.

† One-step, binary logistic regression analysis demonstrated that clusters (OR: 9.7; 95% CI: 1.1–85; *P* = 0.039) and chills (OR 12; 95% CI: 1.8–83; P = 0.010) were two independent features of laboratory-confirmed influenza A.

Likelihood ratios, both positive and negative (Table 3), were calculated for selected symptoms or symptom complexes. Positive likelihood ratio (PLR) is the likelihood for patients with the symptom or the symptom complex to be positive for influenza A compared to those without. Patients with the syndrome of high fever plus cough plus chills plus cluster were 95 (95% CI 12∼750) times more likely to be positive for influenza A than those without this syndrome. Other presentations with high PLR (>5) including high fever plus cough plus chills (24, 95% CI 7.3∼77), chills plus cluster (23, 95% CI 8.1∼64), chills only (9.5, 95% CI 4.3∼21), and high fever plus cough (5.0, 95% CI 2.3∼11).

**Table 3:** Positive likelihood ratio (PLR) and Negative likelihood ratio (NLR) of selected symptoms and syndromes.

Symptom/Syndrome	PLR			NLR		
		95% CI*			95% CI*	
**High fever+cough+chills+cluster**	**95**	12	750	**0.59**	0.36	0.95
**High fever+cough+chills**	**24**	7.3	77	**0.59**	0.37	0.96
**Chills+cluster**	**23**	8.1	64	**0.51**	0.29	0.90
**High fever+chills**	**12**	4.6	31	**0.60**	0.37	0.98
**Chills**	**9.5**	4.3	21	**0.53**	0.30	0.93
**High fever+cough**	**5.0**	2.3	11	**0.64**	0.39	1.03
**High fever+(cough or sorethroat)**	**3.1**	1.5	6.5	**0.68**	0.42	1.09
**High fever**	**2.9**	1.4	6.0	**0.68**	0.42	1.10
**Chilly sensation**	**2.8**	1.7	4.4	**0.44**	0.20	0.98
**Febrile sensation**	**1.9**	1.3	2.7	**0.42**	0.16	1.11
**Cluster**	**1.6**	1.3	2.0	**0.19**	0.03	1.24
**Headache**	**1.4**	0.9	2.1	**0.63**	0.28	1.42
**Cough**	**1.3**	1.0	1.7	**0.48**	0.13	1.73
**Myalgia**	**1.3**	0.8	1.9	**0.70**	0.31	1.58
**Rhinorrhea**	**1.2**	0.9	1.7	**0.67**	0.25	1.81
**Malaise**	**1.1**	0.8	1.6	**0.76**	0.28	2.06
**Sorethroat**	**1.0**	0.7	1.4	**0.95**	0.35	2.59
**Sneezing**	**1.0**	0.6	1.6	**1.00**	0.50	1.99
**Nasal congestion**	**0.8**	0.4	1.3	**1.48**	0.82	2.68

* 95%CI: 95% Confidence Interval.

Negative likelihood ratio (NLR) (Table 3) is the likelihood for patients without the symptom or the symptom complex to be positive for influenza A compared to those with. Patients negative for both cluster and chills were less likely to be with influenza A infection (NLR 0.51; 95% CI 0.29–0.90). It is not a surprise that absence of high fever (NLR 0.68; 95% CI 0.42–1.10) or febrile sensation (NLR 0.42; 95% CI 0.16–1.11) can not exclude influenza A infection.

## Discussion

“Influenza-like Illness” or “flu-like syndrome” has been proposed with a variety of definitions. As revealed with a detailed analysis, flu-like illness is actually nonspecific respiratory illness usually caused not by influenza virus infection but by other respiratory pathogens [2]. However, syndromic approach is still the indispensable clinical practice. It is of great help if grouped symptoms are associated with certain likelihood for the infection. Likelihood of different collections of symptoms may then help decide whether a patient should be sampled for the costly RT-PCR or other confirmatory tests. When influenza A virus infection has become endemic, most cases of respiratory tract symptoms are of the infection. All patients, despite trivial variation of symptoms, have to be managed as with the infection without taking confirmatory test. In a low prevalence setting, the cost-effectiveness of sampling for expensive confirmatory test can be maximized by taking only patients with high likelihood. With increased presence of the virus in the community, the selection criteria can be lowered as the chance of detecting the virus is increased even for patients with symptoms or syndromes of smaller likelihood.

The winter of 2005 in which we performed our prospective study turned out to be a low prevalence flu season. The information of this study is closely relevant to the current situation of low prevalence of the new H1N1 influenza virus worldwide, although the case number of confirmed influenza A virus infection was small. Influenza is a highly contagious infection and spreads out quickly within a household or around a workplace, which results in clustering of cases. It had never been investigated as an indicative factor and did appear to be a very important clue in patient history for identification of influenza A virus infection.

We preferred likelihood ratios over predictive values as the significance of individual symptom or syndrome. Predictive values, profoundly dependent on the prevalence, will not accommodate the rapidly changing epidemiology of influenza infection [5]. Some likelihood ratios are remarkably high in our study. It is probably due to our unbiased patient population. We enrolled all patients with the chief complaint of a new respiratory tract infection. In contrast, the majority of previous studies were based on patient population of flu-like illness [2], [3], [6], [7], [8], [9], [10], [11]. The likelihood ratios of significant symptoms or syndromes for influenza infection were inevitably underestimated.

Although this study was preformed several years ago and case number positive for influenza A infection was very small, we still think this data set is very informative. The major reason is that our study was the first one which indiscriminately invested the expense of molecular testing on a large number of cases not likely to be with influenza A infection. On the contrary, most of the previous studies took selected cases of flu-like illness only and as a result, the distinction of symptomatology between influenza A infections and upper respiratory tract infections of other pathogens was blurred. Currently, as we are concerned about influenza A infection and preparing for the next pandemic, our data set may offer likelihood grading of unbiased precision.

We advocate a tiered approach of syndromic recognition for influenza A virus infection. With low prevalence of infection in the community, in addition to travel history to endemic area, patients with symptoms or syndromes with high likelihood ratio, such as high fever plus chills plus cough, better with clustering of cases in household or workplace are the targets for molecular confirmatory test. With increased prevalence, confirmatory test may be applied to syndromes of less likelihood such as high fever and cough. It should be understood syndromic recognition is not diagnostic and all cases of respiratory tract symptoms have to be managed as with the infection if influenza A virus infection has become endemic.
